# Validation of a 16th Century Traditional Chinese Medicine Use of *Ginkgo biloba* as a Topical Antimicrobial

**DOI:** 10.3389/fmicb.2019.00775

**Published:** 2019-04-16

**Authors:** François Chassagne, Xinyi Huang, James T. Lyles, Cassandra L. Quave

**Affiliations:** ^1^Center for the Study of Human Health, Emory University, Atlanta, GA, United States; ^2^Department of Dermatology, Emory University, Atlanta, GA, United States

**Keywords:** Ginkgo, TCM, ginkgolic acid, skin infections, ESKAPE

## Abstract

In the search for new therapeutic solutions to address an increasing number of multidrug-resistant bacterial pathogens, secondary metabolites from plants have proven to be a rich source of antimicrobial compounds. *Ginkgo biloba*, a tree native to China, has been spread around the world as an ornamental tree. Its seeds have been used as snacks and medical materials in Traditional Chinese Medicine (TCM), while over the last century its leaf extracts emerged as a source of rising pharmaceutical commerce related to brain health in Western medicine. Besides studies on the neuro-protective effects of Ginkgo, its antibacterial activities have gained more attention from researchers in the past decades, though its leaves were the main focus. We reviewed a 16th-century Chinese text, the *Ben Cao Gang Mu* by Li Shi-Zhen, to investigate the ancient prescription of Ginkgo seeds for skin infections. We performed antibacterial assays on various Ginkgo seed extracts against pathogens (*Staphylococcus aureus*, *Cutibacterium acnes*, *Klebsiella pneumoniae*, *Acinetobacter baumannii, Streptococcus pyogenes*) relevant to skin and soft tissue infections (SSTIs). We demonstrate here that Ginkgo seed coats and immature seeds exhibit antibacterial activity against Gram-positive skin pathogens (*C. acnes, S. aureus*, and *S. pyogenes*), and thus validated its use in TCM. We also identified one compound tied to the antibacterial activity observed, ginkgolic acid C15:1, and examine its toxicity to human keratinocytes. These results highlight the relevance of ancient medical texts as leads for the discovery of natural products with antimicrobial activities.

## Introduction

Skin and soft tissue infections (SSTIs) encompass a broad set of conditions encountered frequently in clinical practice, with severities ranging from simple infections, such as subcutaneous abscesses, to life-threatening infections, such as necrotizing fasciitis. Various microbes are involved in skin diseases including *Staphylococcus aureus*, which is a leading cause of SSTIs and is also implicated in atopic dermatitis (Dryden, 2009); *Cutibacterium acnes* (formerly known as *Propionibacterium acnes*) another Gram-positive bacterium, which can cause acne vulgaris, blepharitis, dandruff and psoriasis (Findley and Grice, 2014); *Streptococcus pyogenes*, a group A streptococci, which cause impetigo, erysipelas, and necrotizing fasciitis (Bisno and Stevens, 1996); as well as *Acinetobacter baumannii*, *Klebsiella pneumoniae*, and *Pseudomonas aeruginosa –* three Gram-negative bacteria that are implicated in burn wound infections and various types of SSTIs (e.g., necrotizing fasciitis for *A. baumannii* and hot tub folliculitis for *P. aeruginosa*) (Chim et al., 2007; Sensenig et al., 2012). Over the past decade, a rise in antibiotic resistant strains of these species have been reported. *A. baumannii*, *K. pneumonia*, and *S. aureus* are included in the list of six “ESKAPE” pathogens (*Enterococcus faecium*, *S. aureus*, *K. pneumoniae*, *A. baumannii*, *P. aeruginosa*, and *Enterobacter* spp.). These six pathogens show growing number of strains with multidrug-resistant profile which are responsible for high morbidity and mortality in patients (Boucher et al., 2009). For example, methicillin-resistant *S. aureus* (MRSA) is estimated to cause between 11,000 and 21,000 deaths annually in the United States (Dantes et al., 2013). Moreover, more than 50% of *C. acnes* strains have been reported to be resistant to topical macrolides in many countries (Walsh et al., 2016). Overall, the treatment of these infections has become increasingly challenging and there is a rising need for new antibacterial agents.

Traditional Chinese Medicine (TCM) remains one of the oldest healing systems and is believed to have originated around 3,000 years ago (Jiuzhang and Lei, 2009). Chinese Materia Medica (*Ben Cao*) refers to the botanical, mineral and zoological substances used in TCM, and its oldest record (*Wu Shi Er Bing Fang*) dates back to approximately 1100 BCE and includes 247 natural agents and roughly 150 combinatorial drug formulae (Wu, 2005). Several medicinal books on materia medica have been produced, including the well-known *Ben Cao Gang Mu* (The Grand Compendium of Materia Medica) of Li Shi-Zhen, published in 1587 CE. This book includes 1,892 species and about 11,000 combinatorial formulae. It represents not merely an herbal medicine record, but also a historical guide to medicine (not limited to plants), including cultural factors, past annotations from famous physicians, remedies to diseases, and prescriptions (Yuqun, 2008; Ji et al., 2009).

In the TCM system, skin diseases are usually regarded as manifestations of internal diseases. Most of the TCM prescriptions for skin disorders are multi-ingredient recipes which combine several plants that would be ineffective if used in isolation. The composition of these mixtures of herbs varies individually depending on the type of skin disorders and the pathologies associated. Remedies are prepared in the form of decoctions and taken orally for several weeks (Sheehan and Atherton, 1992; Bedi and Shenefelt, 2002; Hon et al., 2011). Topical preparations are also used in TCM, and their composition is often much simpler, even restricted to a single ingredient in some cases (Koo and Desai, 2003). From a pharmacological point of view, the remedies composed of a single plant species are easier to study and thus could represent a gateway to the scientific validation of TCMs. Moreover, the herb used could be the source of new, potent antibacterial compounds.

*Ginkgo biloba* L. of the Ginkgoaceae family is a deciduous tree (20–40 meters height) native to eastern China and is considered a “living fossil” since it probably originated 200 million years ago (Singh et al., 2008). It was first recorded as a medicinal plant in the Chinese Materia Medica *Shen Nong Ben Cao Jing* approximately 2,000 years ago, and only the seeds were reported to be used as medicine (Del Tredici, 1991). Much later, the leaves of *G. biloba* were cited for the treatment of heart and lung diseases in TCM (Mahady, 2001), and this part of plant attracted most attention from outside China. In 1964, an extract of Ginkgo leaves (EGb 761) was introduced into Western medical practice, and since that time these standardized extracts (commercially available as Tanakan^^®^^, Tebonin^^®^^, Rokan^^®^^) have been used worldwide for the treatment of mild to moderate age-associated memory impairment, dementia and peripheral vascular diseases (Ude et al., 2013). Although Ginkgo seeds have long played a key role in TCM, most of the current research has focused on the properties of leaf extracts and, to the best of our knowledge, no studies aimed to evaluate the seeds for antimicrobial activity against skin pathogens.

The aims of our study were to first to detail the ancient prescription of Ginkgo seeds for skin disorders in TCM by reviewing the Chinese Materia Medica *Ben Cao Gang Mu*, and second, to validate this traditional use by investigating and comparing the antibacterial activities of different Ginkgo extract formulations (from seed kernels, seed coats, immature seed, leaves, and branches) as well as various Ginkgo derived compounds on bacterial strains commonly found in skin disorders.

## Materials and Methods

### Historical Text Review

**FIGURE 1:**
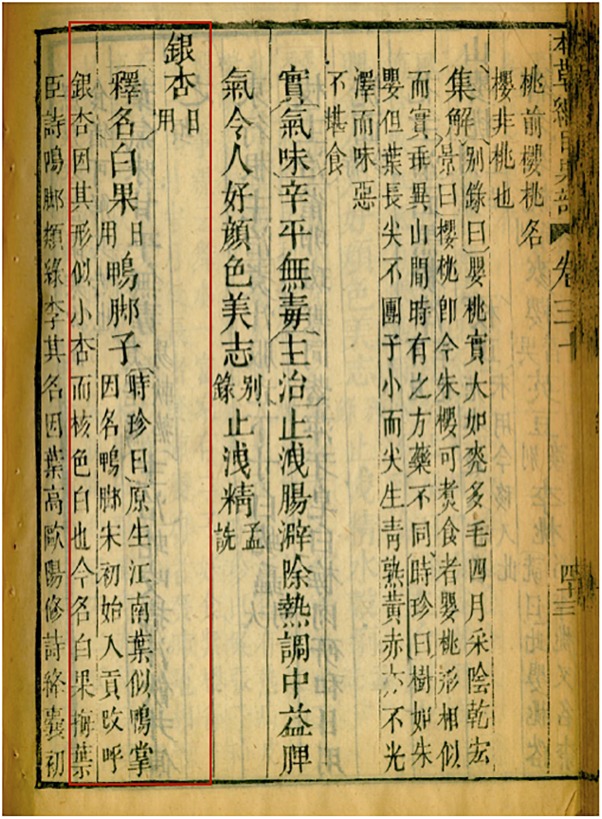
*Ben Cao Gang Mu* text. A block-printed copy made in China, 1826, stored in Emory University Pitts Theology Library. The page shown here is the first page of the Ginkgo record and the relevant text is highlighted with a red box. It translates to: “Ginkgo (yin xing) (daily use). Explanation of its name [alternate name] White Fruit (Bai guo) (daily use) [other name] Duck Foot.” Shizhen commented: “[Ginkgo] originated in Jiangnan [region south of Yangtze River]. Its leaves look like duck flipper, so it got its nickname: the Duck Foot. Starting from Song Dynasty, Ginkgo was a tribute to royal families and was re-named the silver apricot (yin xing), because it looks like small apricot and its inner nutshell is white. Nowadays, it is called the white fruit (bai guo).”

The Compendium of Materia Medica *Ben Cao Gang Mu* (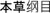) is a collection of previous annotations and summarized over 40 earlier pharmacopeia and more than 300 medical texts. A block-printed copy of the Compendium of Materia Medica *Ben Cao Gang Mu* is stored at the Emory University Pitts Theology Library in Atlanta, United States and was used for our historical review. Since this copy was printed in 1826 in China and the original text was produced in 1587, we also used two other original historical texts (i.e., Simple remedies for emergencies, *Jiu ji yi fang* (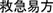) and General remedies, *Pu ji fang* (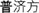) included in the *Ben Cao Gang Mu* and also found separately at the Emory University Pitts Theology Library, to check for any mistakes in the block-printed copy. A thorough examination of each reference related to skin diseases for *Ginkgo biloba* (known as *yin xing* 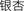 in Chinese) was performed in the *Ben Cao Gang Mu* (Figure 1). The whole record was translated in English by the co-first author (X. Huang) (Supplementary Figure S1).

### Gingko Materials

**FIGURE 2:**
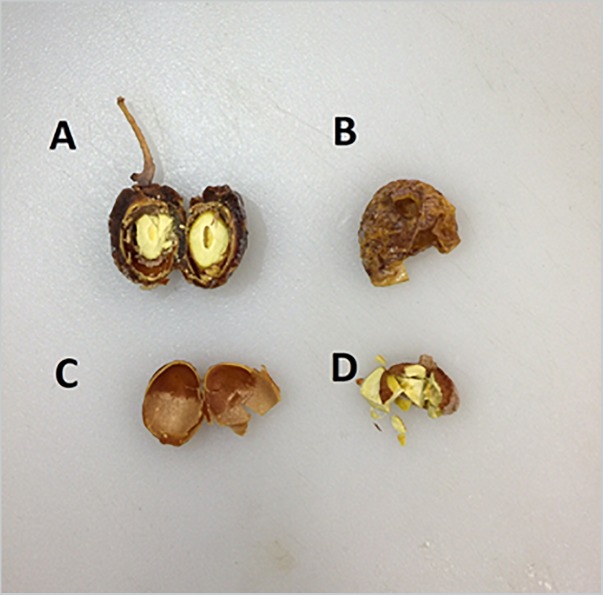
Dissection of Ginkgo seed. **(A)** Cross section of whole Ginkgo seed. **(B)** Testa, or seed coats. **(C)** Nutshell [not included in assay]. **(D)** Seed nuts, or kernel.

Ginkgo tree samples were collected from a female and a male tree on Emory University Campus in September and October 2015, while the leaves were still green. Voucher specimens (GEO20494, GEO20496, and GEO20497) were deposited at the Emory University Herbarium (GEO) in Atlanta, GA, United States, where they were also digitized and made available for viewing on the SERNEC portal (SERNEC Portal, 2018). Additional fresh seeds were purchased from a local farmer’s market: Buford Highway Farmers Market (5600 Buford Hwy NE, Atlanta, GA 30340). Samples were separated by gender, then by different parts: leaves, branches, seeds and immature whole seeds. Mature seeds (diameter larger than 1.5 cm with testa/integument soft to touch) were separated into seed coat (testa/integument) and seed nut (kernel) (Figure 2). Dry samples were prepared by dehydrating the plant materials in drying cabinet for at least 5 days and grinding into fine powder with a Thomas Wiley Mill using a 2 mm mesh size (Thomas Scientific, Swedesboro, NJ, United States). Fresh-frozen materials were prepared by freezing the plant materials forthwith at −20°C and being stored until extraction.

### Chemical Standards

Nine chemicals known to be present in *Ginkgo biloba* were obtained from various suppliers: bilobalide (Fisher Scientific, Hampton, NH, United States), ginkgolide A (LKT Laboratories, St. Paul, MN, United States), ginkgolic acid C15:1, ginkgolide B, isorhamnetin, and quercetin (Cayman Chemical Co., Ann Arbor, MI, United States), ginkgotoxin and rutin (Sigma Aldrich, Saint-Louis, MO, United States), kaempferol (Enzo Life Sciences Inc., Farmingdale, NY, United States) (Supplementary Figure S2).

### Plant Extraction

According to the *Ben Cao Gang Mu*, the topical usage of Ginkgo seed involved a delivery media of water, alcohol, and oil. Thus, extractions of Ginkgo seeds were processed with water, 80% ethanol, and rape seed oil. A decoction extraction (boiling plant material in water to extract chemical substances) was performed to Ginkgo leaves, since in *Quan guo zhong cao yao hui bian*, the *Summary of Chinese Herbal Medicine*, Ginkgo leaf boiled in water was a treatment for enteritis in children (Yu et al., 1996).

Samples were extracted with solvent at a ratio of 1 g:10 mL, regardless of sample type, solvent type or extraction method. For macerated extractions, plant materials were mixed with 80% ethanol for at least 72 h with daily agitation; macerations were repeated one time, and the marcs (residue that remains following the extraction) combined. For decoctions, plant materials were heated with dH_2_O on a hot plate with a stir bar for 25 min at 100°C; the decoction products were then filtered after cooling to room temperature. For sonication assisted extractions, plant materials were mixed with dH_2_O and sonicated (at room temperature) for two successive 20 min periods, and the filtrate combined. For oil infusions, plant materials were mixed with rape seed oil over a 3 month period with daily agitation. Filtration of ethanolic extracts was performed under vacuum using coarse (Fisher brand P8^^®^^, Fisher Scientific) and then fine (Fisher brand P2^^®^^) filter papers. For aqueous extracts, materials were filtered through cheese cloth, then through coarse (Whatman 113) filter paper, the filtrate was centrifuged at 657 rcf for 10 min, then filtered through fine (Ahlstrom wet strength) filter paper. For oil extracts, materials were filtered through cheese cloth, centrifuged at 657 rcf for 5 min, and then decanted. With the exception of the oil infusion, all extract solvents were evaporated at < 58 atm pressure at 38°C with rotary evaporators. Extracts were re-suspended with dH_2_O and lyophilized, and then stored at −20°C until being tested. For biological assays, ethanol extracts were dissolved in 100% DMSO at a concentration of 10 mg/mL, aqueous extracts were dissolved in 50% DMSO_(aq)_ at 10 mg/mL, and the oil infusion was prepared at 10% v/v in 100% DMSO to achieve a test range of 0.04–0.512% v/v.

### Bacterial Strains and Culture Conditions

Twelve strains from five different bacterial species were used for this survey including *Acinetobacter baumannii* (EU-25, EU-26, EU-27), *Klebsiella pneumoniae* (EU-32, CDC16, CDC49), *Cutibacterium acnes* (ATC6919), *Streptococcus pyogenes* (EU-20, EU-21), and three strains of *Staphylococcus aureus* (UAMS-1, LAC, AH430). Details regarding their antibiotic resistance profiles can be found in Supplementary Table S1.

All strains (except *C. acnes* and *S. pyogenes*) were streaked from freezer stock onto tryptic soy agar (TSA) plates and incubated at 37 °C overnight before making overnight liquid cultures in cation-adjusted Mueller-Hinton broth (CAMHB) or tryptic soy broth (TSB). *S. pyogenes* strains were streaked on TSA + 5% defibrinated sheep blood and incubated at 37 °C at 5% CO_2_, and liquid cultures were made in Brain Heart Infusion (BHI). *C. acnes* strains were treated as previously described (Nelson et al., 2016).

### Growth Inhibition Assays

The minimum inhibitory concentration (MIC) for extracts was determined according to the CLSI guidelines (CLSI, 2013). For *C. acnes*, we followed previously described methods with some modifications (Tsai et al., 2010; Nelson et al., 2016). Concentrations of the overnight culture was determined using a Cytation 3 multimode plate reader (Biotek^^®^^, Winooski, VT, United States) by optical density (OD_600__nm_), then was standardized to 5 × 10^5^ CFU/mL with CAMHB media (or to 5 × 10^7^ CFU/mL in BHI with 1% dextrose for *C. acnes*). Assays were performed in 96-well plates (Greiner Bio-One International, CELLSTAR^^®^^ 655-185). Vehicles (100% DMSO or 50% DMSO) and antibiotics (gentamicin and tetracycline for *A. baumannii* and *K. pneumoniae*; ampicillin and vancomycin for *S. aureus*; erythromycin and clindamycin for *C. acnes;* ampicillin and erythromycin for *S. pyogenes*) were tested as controls for each test performed. The final total percent DMSO in the well volume was <5% for all assays. The concentrations of extract/control tested varied from 512 to 4 μg/mL in serial dilution, and for antibiotics/standards 64–0.5 μg/mL (4–0.03125 μg/mL for Ery and Cln only). All concentrations were tested in triplicate and all tests were repeated at least once on a separate day. The OD_600__nm_ of plates was read at 0 h and after 18 h of static incubation with humidity at 37°C (22 hrs for *A. baumannii*; 24 h for *S. pyogenes*; 72 h and anaerobic for *C. acnes*). The percent inhibition of bacteria growth was calculated using the formula: (1 – (ΔOD_extract_/ΔOD_vehicle_))^∗^100 (Quave et al., 2008). The IC_50_ and MIC were determined as the concentration in which 50 and 90% of bacteria growth were inhibited, respectively.

### Biofilm Inhibition Assays

All extracts were examined for biofilm inhibitory activity in four species: *A. baumannii*, *K. pneumoniae*, *C. acnes*, and *S. aureus* using previously described methods (Quave et al., 2012; Nelson et al., 2016; Muhs et al., 2017). For *S. aureus*, we used the well-characterized methicillin-sensitive *Staphylococcus aureus* (MSSA) osteomyelitis strain (UAMS-1), and its isogenic ΔsarA mutant, UAMS-929, which has a biofilm deficient phenotype and serves as a positive control. We also included the natural product-based anti-biofilm composition “220D-F2,” which has been shown to inhibit biofilm formation in *Staphylococcus aureus* (Quave et al., 2012). For *A. baumannii*, *K. pneumoniae*, and *C. acnes*, we used antibiotics as positive controls (i.e., gentamicin and tetracycline for *A. baumannii* and *K. pneumoniae*; erythromycin and clindamycin for *C. acnes*). Briefly, following inoculation and addition of appropriate media (containing extract or vehicle alone), 96-well plates (Falcon^^®^^ 35–1172) were incubated for 22 h at 37°C. The wells were gently washed with phosphate-buffered saline (PBS), fixed with ethanol (for *S. aureus* only), stained with crystal violet, rinsed in tap water, and the stain eluted into ethanol and transferred to a new plate prior to quantification of the elute at an OD_595nm_ with a Cytation 3 multimode plate reader. MBEC_50_ (used for *C. acnes*) refers to the minimum concentration necessary to eradicate attached biofilm. MBIC_50_ and MBIC_90_ values refer to the minimum concentration necessary to achieve ≥50 and ≥90 inhibition of biofilm formation, respectively.

### Cytotoxicity Assays

Human immortalized keratinocytes (HaCaTs) were used to evaluate the potential skin toxicity of each Ginkgo extract, by employing a lactase dehydrogenase (LDH) cytotoxicity assay (G-Biosciences, St. Louis, MO, United States) as previously described (Quave et al., 2015). Extracts were tested at a concentration from 4 to 512 μg/mL by twofold serial dilution. All concentrations were tested in triplicate. Plates were read by a Cytation3 multimode plate reader at OD_490__nm_.

To determine the cytotoxicity of extracts, percentage cytotoxicity was calculated using the following equation: (1 – (OD_extract_ – OD_spontaneous_)/OD_max_))^∗^100, where OD_max_ is the OD_490__nm_ read for wells treated with lysis buffer after incubation to achieve maximum of cell lysis, and OD_spontaneous_ is the OD_490__nm_ read for wells without treatment. The Therapeutic Index (TI) was calculated as a ratio of the TD_50_ (Toxic dose for cytotoxicity at IC_50_) and ED_50_ (effective dose for growth inhibition at IC_50_): = $\frac{{\text{TD}}_{50}}{{\text{ED}}_{50}}$.

### Chemical Characterization by TLC and HPLC

Preliminary characterization of the extracts was pursued by Thin Layer Chromatography (TLC) and High-Performance Liquid Chromatography (HPLC). TLC was employed to look for presence of bilobalide, ginkgolide A, ginkgolide B as described in the American Herbal Pharmacopeia (Upton, 2003), while HPLC methods were used for detecting presence of quercetin, kaempferol, isorhamnetin, rutin, and ginkgolic acid C15:1 using the method described by Dubber and Kanfer (2004). The HPLC analysis was performed on an Agilent 1260 Infinity system equipped with a diode array detector running OpenLab CDS ChemStation (Agilent Technologies, Santa Clara, CA, United States) with an Agilent^^®^^ ZORBAX Eclipse XDB-C18 (250 mm × 4.6 mm, 5 μm) column with compatible guard column at a column temperature of 45°C. Mobile phase reagents were HPLC-grade and purchased from Fisher Scientific, except for the Type 1 water, which was obtained from an EMD Millipore MILLI-Q water system. The mobile phase consisted of a gradient elution of water in 0.3% formic acid (A) and acetonitrile (B) at a flow rate of 1 mL/min. Initial conditions were 98:2 (A:B), changing to 85:15 (A:B) at 20 min, then to 75:25 (A:B) at 50 min and reaching 0:100 (A:B) at 100 min; this was maintained until 115 min, and then the column was returned to initial conditions 98:2 (A:B) and held for 15 min. Samples were prepared in either 90:10 ACN: water or 35:35:30 methanol:water:ethyl acetate depending on the solubility of extracts and 20 μL HPLC injections were made. Chromatograms were monitored at 245 nm and compounds were identified by comparing the chromatograms of the Ginkgo extracts to those obtained from the standard compounds.

### Quantification Analysis by HPLC

Quantification of ginkgolic acid C15:1 in the most active extracts which either exhibited a MIC_50_, MBIC_50_ MBEC_50_, was pursued by HPLC with the same instrument using an Agilent^^®^^ Poroshell 120 EC-C18 (50 mm × 4.6 mm, 2.7 μm) (Supplementary Figure S3). The gradient had initial conditions of 75:25 (A:B), changing to 0:100 (A:B) at 2 min, then the column was returned to initial conditions at 8 min and held for 4 min. Samples were prepared in 35:35:30 methanol:water:ethyl acetate, and triplicate 20 μL injections were made. Concentration was calculated as previously described (Lyles et al., 2017). The limits of detection (LOD) and quantification (LOQ) were calculated from the standard curves at 3.3σ and 10σ, respectively (ICH, 2005).

### Statistical Analysis

GraphPad Prism software v.7 (GraphPad software, La Jolla, CA, United States) was used to conduct statistical tests. Data were analyzed with a Student’s *t*-test (unpaired, 2-tailed) when two groups (numerical data) were compared, or with ANOVA analysis when more than two groups (numerical data) were studied. The correlation between the antimicrobial score and the concentration of ginkgolic acid C15:1 in each active Ginkgo extract was explored with a regression analysis. In all cases, *P* < 0.05 was considered statistically significant.

## Results

### Ethnomedicinal Records of *Ginkgo biloba*

In the *Ben Cao Gang Mu*, Li Shi-Zhen used the term “*bai guo*” (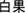, or Ginkgo seed) to refer to *Ginkgo biloba* tree, and did not refer to any other part of the plants. In total, 17 traditional uses were reported in the book, including 8 for skin disorders such as chapped hands and feet, rosacea, patches and nodules on the face and scalp, genital ulcers, crab louse-induced itchiness, dog bite wound abscess, mastitis, bullae or pustules. Of these eight prescriptions, three were already reported in previous historical texts while the five others were original prescriptions. In the methods of preparation, Li Shi-Zhen mentioned applying the paste of raw gingko kernels to the affected area, or rubbing (sliced open) raw kernels on it. The paste can be prepared by chewing, crushing, crushing with wine or distillery draff, or crushing it after being immersed in oil for years.

### Growth Inhibitory Activity of Ginkgo Extracts

A total of 27 extracts were tested including: 11 dried extracts and 16 fresh-frozen extracts; 18 extracts from female trees and 9 from male trees; 13 ethanolic extracts, 9 extracts sonicated in water, 4 extracts decocted in water, and 1 extract macerated in rape seed oil; as well as 12 extracts from leaves, 6 extracts from branches, 6 extracts from seed, 2 extracts from seed coats, and 1 extract from immature seeds.

Out of these 27 extracts tested, 18 showed growth inhibitory activity against at least one bacterial strain at a concentration of 512 μg/mL or lower (Table 1). Moreover, 6, 5, 13, and 11 extracts exhibited an IC_50_ less than 100 μg/mL on *C. acnes*, *S. aureus* UAMS-1, *S. pyogenes* EU20, and *S. pyogenes* EU21, respectively, and thus can be considered as “active” as defined by Cos et al. (2006). Our extracts were particularly active against *S. pyogenes* with 9 and 5 extracts having a MIC below 8 μg/mL for *S. pyogenes* EU20 and EU21, respectively.

**Table 1:** MICs and MBICs of extracts from different Gingko biloba plant tissues.

Part^A^	Stor^B^	Ext^C^	ID^D^	*A. baumannii*			*K. pneumoniae*			*C. acnes*		*S. aureus*				*S. pyogenes*		Cyt^F^
												
				EU25	EU26	EU27	EU32	CDC16	CDC49	ATCC6919		UAMS-1		AH430	LAC^E^	EU20	EU21	
				IC_50_	IC_50_	IC_50_	IC_50_	IC_50_	IC_50_	IC_50_/MIC	MBEC_50_	IC_50_/MIC	MBIC_50/90_	IC_50_/MIC	IC_50_/MIC	IC_50_/MIC	IC_50_/MIC	IC_50_
**FEMALE TREE (ACCESSION N.: GEO20494)**																		
IS	FF	ME	678	–	–	–	512	–	–	64/64	256	64/−	256/512	128/−	128/512	4/8	/8	–
Le	Dry	ME	660	–	–	–	512	–	–	256/256	–	128/−	–	512/512	256/−	16/32	/32	512
	FF	ME	671	512	512	–	–	–	–	256/512	–	256/512	–	512/−	512/−	/16	/32	–
	Dry	D	661	–	–	–	–	–	–	−/−	–	−/−	–	−/−	−/−	128/−	−/−	–
	FF	D	672	512	–	–	–	–	–	−/−	–	−/−	–	−/−	−/−	−/−	−/−	–
SC	Dry	ME	665	–	–	–	–	–	–	64/64	–	32/−	64/−	128/−	128/−	/8	/8	512
	FF	ME	677	–	–	–	512	–	–	64/512	–	−/−	32/512	128/−	128/−	4/8	8/16	–
SN	Dry	ME	663	–	–	–	–	–	–	−/−	–	−/−	256/512	512/−	−/−	64/−	−/−	–
	FF	ME	674	–	–	–	512	–	–	−/−	–	−/−	32/−	−/−	−/−	32/128	128/−	–
	FF	SW	689	–	–	–	512	–	–	−/−	–	256/−	–	128/−	−/−	−/−	−/−	–
	FF	MO	676	–	–	0.256	–	–	–	−/−	–	−/−	0.128/−	0.512/−	−/−	0.128/−	−/−	–
WB	Dry	ME	666	512	512	128	512	–	512	128/256	–	64/−	16/128	−/−	512/−	/8	/16	512
	FF	ME	679	–	–	–	–	–	–	64/64	–	64/−	–	128/−	−/−	/8	/16	512
	FF	SW	687	512	–	–	–	–	–	−/−	–	512/−	–	512/−	512/−	128/−	−/−	512
**MALE TREE (ACCESSION N.: GEO20496; GEO20497)**																		
Le	Dry	ME	667	512	512	–	–	–	512	128/256	–	−/−	–	256/−	512/−	4/8	/16	512
	FF	ME	680	512	512	128	512	–	–	128/256	–	128/−	–	512/−	256/−	4/8	/8	512
	Dry	D	668	–	–	–	–	–	–	−/−	–	512/−	–	−/−	512/−	/128	−/−	–
	FF	D	681	–	–	–	–	–	–	−/−	–	−/−	–	−/−	−/−	−/−	−/−	–
WB	Dry	ME	670	–	–	–	–	–	–	64/64	–	−/−	32/512	128/−	256/−	/4	4/8	256
	FF	ME	683	–	–	–	–	–	–	16/16	–	32/−	32/256	64/−	32/128	< 2/ ≤ 2	<2/ ≤ 2	128
**COMPOUND STANDARDS**																												
Ginkgolic acid C15:1				–	–	–	32	–	–	2/4	16	2/4	–	4/8	1/2	0.5/1	/1	16
Ginkgolide A				–	–	–	–	–	–	−/−	–	−/−	–	−/−	−/−	–	–	–
Ginkgolide B				–	–	–	–	–	–	−/−	–	−/−	–	−/−	−/−	–	–	–
Ginkgotoxin				–	–	–	–	–	–	−/−	–	−/−	–	−/−	−/−	–	–	–
Bilobalide				–	–	–	–	–	–	−/−	–	−/−	–	−/−	−/−	–	–	–
Rutin				–	–	–	–	–	–	−/−	–	−/−	–	−/−	−/−	–	–	–
Quercetin				–	–	–	–	–	–	−/−	–	32/−	–	16/−	16/−	–	–	–
Isorhamnetin				–	–	–	–	–	–	−/−	–	−/−	–	−/−	64/−	–	–	–
Kaempferol				–	–	–	–	–	–	−/−	–	64/−	–	−/−	16/−	–	–	–
**Antibiotic Controls**																		
Clindamycin				NT	NT	NT	NT	NT	NT	0.12/0.25	8	< 0.25	NT	< 0.25	<0.25	< 0.25	<0.25	NT
Erythromycin				NT	NT	NT	NT	NT	NT	0.03/0.06	NT	< 0.25	NT	< 0.5	<0.25	< 0.12	<0.12	NT
Gentamycin				< 1	> 16	>16	0.5	0.25	> 16	NT	NT	< 0.5	NT	< 0.5	<0.5	NT	NT	NT
Tetracycline				< 2	> 16	1	2	16	> 32	NT	NT	< 1	NT	< 1	<1	< 0.25	<0.25	NT
Ampicillin				< 4	<8	> 32	>32	> 32	>32	/0.02	NT	8/64	NT	0.5/0.5	4/64	< 0.25	<0.25	NT
Vancomycin				NT	NT	NT	NT	NT	NT	NT	NT	1/1	NT	0.5/1	0.5/1	0.25	0.25	NT

*^A^Plant part: Le, Leaves; IS, immature seed nut; SC, seed coat; SN, seed nut; WB, wood (from branches). ^B^Storage of plant materials prior to extraction. Dry, Air dried; FF, fresh-frozen. ^C^Extraction process. D, decoction in water (boiled in water × 25 min.); ME, maceration in 80% ethanol; MO, maceration in rapeseed oil; SW, sonicated in water at room temperature. Only SW extracts with antibacterial activity are shown in the table. ^D^ID, Extract identification number in the Quave Natural Products Library (QNPL). ^E^LAC (Los Angeles Clone) bacterial strain is also called AH1263. “-”, No activity detected at the test range (max concentration of 512 μg/mL for all plant extracts; max of 64 μg/mL for all single compounds). All values are reported in μg/mL, with exception of the oil macerate preparation, which is represented as % volume oil/well volume. “NT”, Not tested. ^F^Cytotoxicity activity on HaCat cell lines.*

Among the nine Ginkgo standard compounds tested, only four demonstrated antibacterial activity, with ginkgolic acid C15:1 (the most abundant of the six known ginkgolic acids in *Ginkgo biloba*) exhibiting the highest level of antibacterial activity.

By comparing growth inhibitory activity at 512 μg/mL between Ginkgo samples, we found statistically significant differences between percent inhibition of the four extraction methods used (i.e., ethanol maceration, water sonication, water decoction, and oil infusion), as well as significant differences between tree parts (i.e., branches, leaves, seed, seed coats, and immature seeds). However, we did not find significant differences between the percent inhibition of extracts that were from male and female trees, or the extracts that were dry or fresh-frozen before testing (Supplementary Figure S4).

We then compared the effect of extraction methodologies on the inhibitory activities of Ginkgo extracts for six bacterial strains. As a general trend for all 6 strains, ethanolic extracts exhibited inhibitory activities that were significantly greater than extracts made any other way in the study (Supplementary Figure S5).

**FIGURE 3:**
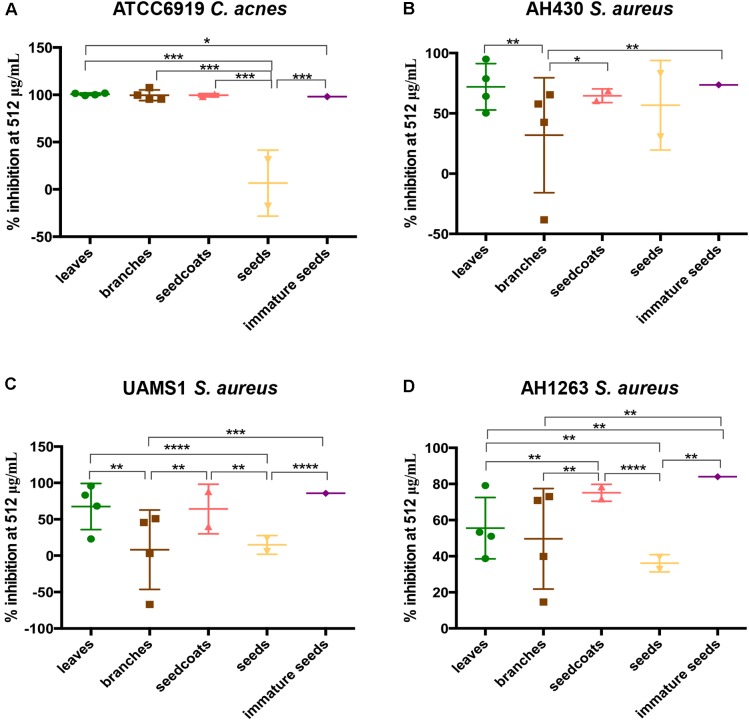
Comparison of plant tissues in MIC assays. Percent inhibition of tree parts on growth activity of *C. acnes* ATC6919 **(A)**, *S. aureus* AH430 **(B)**, *S. aureus* UAMS1 **(C)**, and *S. aureus* AH1263 (LAC) **(D)** at 512 μg/mL. The significance levels of each comparison were based on the student’s *t*-test results on the right of each scatter plots. ^∗^*p* ≤ 0.05, ^∗∗^*p* ≤ 0.01, ^∗∗∗^*p* ≤ 0.0001, ^∗∗∗∗^*p* ≤ 0.00001.

Regarding the effect of tree parts on the inhibitory activities of Ginkgo samples for each bacterial strain, we found different results depending on the bacterial species tested (*A. baumannii* and *K. pneumoniae* were not included in the analysis due to the low level of antibacterial activities of Ginkgo samples against these species). For *C. acnes*, inhibitory activities of seed nuts were significantly lower than for other tree parts. For *S. aureus* UAMS-1, inhibitory activity of immature seeds was significantly higher than branches and seed coats, while seed coats exhibited higher inhibitory activities compared to branches and seed nuts. For *S. aureus* AH430, inhibitory activities of seed coats and immature seeds were higher than those of branches. Finally, for *S. aureus* LAC, immature seeds and seed coats exhibited higher inhibitory activities than leaves, branches and seed nuts (Figure 3).

### Anti-biofilm Activity of Ginkgo Extracts

**FIGURE 4:**
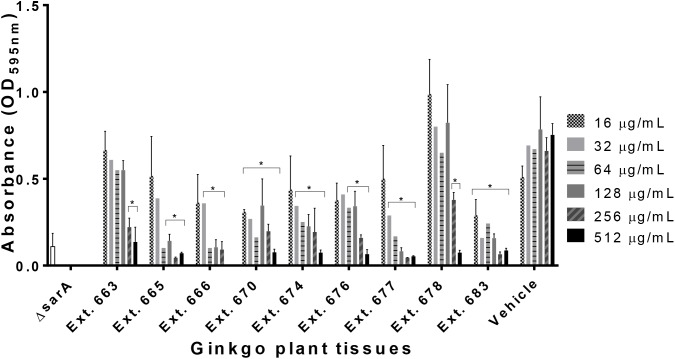
Anti-biofilm activity of Ginkgo plant tissues against *S. aureus* UAMS-1. ΔsarA mutant represents the positive control as described previously (Quave et al., 2012). Statistical significance in comparison to the vehicle treated is denoted as ^∗^*P* < 0.05.

Out of the 27 extracts tested, 9 samples showed biofilm inhibitory activity (Table 1). None of the extracts exhibited anti-biofilm activity on *A. baumannii* (EU27) and *K. pneumoniae* (EU32), only one extract had activity on *C. acnes* biofilm, and nine showed biofilm inhibitory activity on *S. aureus* UAMS-1 with IC_50_ ranging from 16 to 256 μg/mL (Figure 4). Regarding the nine Ginkgo standards tested, only ginkgolic acid C15:1 showed anti-biofilm activity and exhibited an IC_50_ of 16 μg/mL on *C. acnes* biofilm eradication.

### HaCaT Cytotoxicity Assay

Extracts with IC_50_ values detected for bacterial growth were examined for cytotoxicity against an immortalized line of human keratinocytes in order to determine whether the observed antibacterial activity was due to specific antibacterial action or general toxicity (Table 1). Of the 17 Ginkgo extracts tested, 9 exhibited cytotoxic activity against HaCaT cell lines, with IC_50_ ranging from 128 to 512 μg/mL. The two most cytotoxic samples were branch extracts. Regarding the 9 chemical standards tested, only ginkgolic acid C15:1 exhibited cytotoxicity against human keratinocytes, with an IC_50_ of 16 μg/mL.

By comparing the cytotoxicity of active extracts at 512 μg/mL, we found statistically significant differences between the seeds and immature seeds as compared to others part of plants (i.e., leaves, branches, and seed coats) (Supplementary Figure S6). Immature seeds and seed coats also exhibited the highest TI for *C. acnes* and *S. aureus* with a value ≥ 16.

### Chemical Characterization and Quantification of Ginkgolic Acid C15:1

**FIGURE 5:**
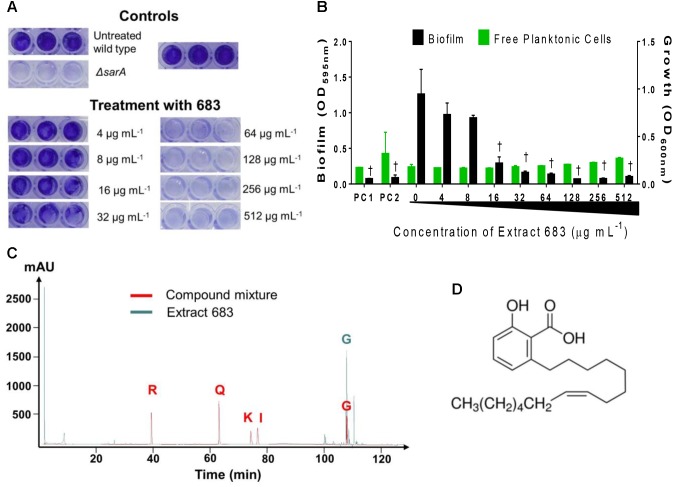
Biofilm inhibition assay on *S. aureus* UAMS-1 and identification of five Ginkgo standard compounds for the most active Ginkgo extract (no. 683). **(A)** Images of crystal violet stained biofilm in 96-well plates. USA 200 isolate UAMS-1 and its isogenic sarA mutant (UAMS-929) were used in the biofilm assay. **(B)** The optical density (OD_595__nm_) of the crystal violet eluent is plotted with the OD_600__nm_ for planktonic cells, measured by transfer of the well supernatants to a new 96-well plate. Two different positive controls (PC) are represented in the figure: PC1 = 220D-F2 extract; PC2 = ΔsarA mutant as previously described (Quave et al., 2012). Statistical significance in comparison to the vehicle treated wild type control is denoted as ^†^*P* < 0.001. **(C)** HPLC chromatograms at 245 nm of Ginkgo standard compounds (i.e., quercetin 0.4 mg/mL, isorhamnetin 0.2 mg/mL, kaempferol 0.4 mg/mL, rutin 0.4 mg/mL, ginkgolic acid C15:1 0.4 mg/mL) and Ginkgo extract no. 683 (10 mg/mL) are shown in red and green line, respectively. **(D)** Chemical structure of ginkgolic acid C15:1.

In order to provide basic chemical characterization of each active sample, we used a panel of standard Ginkgo chemicals. The 17 active Ginkgo extracts were examined by TLC and HPLC for the presence of 8 compounds: ginkgolide A, ginkgolide B, ginkgolic acid C15:1, bilobalide, quercetin, kaempferol, isorhamnetin, and rutin by comparison with authentic standards (Table 2). Regarding the presence of standard compounds showing antibacterial activities in our study, none of the Ginkgo extracts contained quercetin, isorhamnetin or kaempferol, whereas all ethanolic Ginkgo extracts contained ginkgolic acid C15:1 (Figure 5). We quantified the ginkgolic acid C15:1 in each ethanolic Ginkgo sample, and the concentration in the 13 active Ginkgo extracts ranged from 1.31 to 84.79 mg/g of dried plant material (Supplementary Table S2). All quantifications were above the limits of detection (LOD = 0.11 μg) and limits of quantification (LOQ = 0.33 μg) (Supplementary Figure S7).

**Table 2:** Chemical characterization of the active extracts and quantification of ginkgolic acid C15:1.

Part^A^	Stor^B^	Ext^C^	ID	(1)	(2)	(3)	(4)	(5)	(6)	(7)	(8)^D^
**FEMALE TREE**											
IS	FF	ME	678	+	−	−	−	−	−	−	63.34 ± 3.44
Le	Dry	ME	660	+	−	+	−	−	−	+	17.33 ± 0.85
	FF	ME	671	−	+	−	−	−	−	+	10.09 ± 0.22
SC	Dry	ME	665	−	−	−	−	−	−	−	29.88 ± 1.38
	FF	ME	677	+	−	−	−	−	−	−	48.59 ± 0.16
SN	Dry	ME	663	−	−	−	−	−	−	−	1.31 ± 0.06
	FF	ME	674	−	−	−	−	−	−	−	4.66 ± 0.05
	FF	SW	689	+	−	−	−	−	−	−	−
	FF	MO	676	NT	NT	NT	NT	NT	NT	NT	NT
WB	Dry	ME	666	+	−	+	−	−	+	−	27.24 ± 0.25
	FF	ME	679	−	+	−	−	−	−	−	31.56 ± 0.79
	FF	SW	687	−	+	−	−	−	−	−	−
**MALE TREE**											
Le	Dry	ME	667	−	−	+	−	−	+	+	33.27 ± 2.44
	FF	ME	680	+	−	−	−	−	−	+	25.93 ± 0.81
	Dry	D	668	−	−	−	−	−	−	+	−
WB	Dry	ME	670	+	−	+	−	−	−	−	65.99 ± 0.57
	FF	ME	683	−	+	+	−	−	−	−	84.79 ± 0.94

*^A^Plant part: Le, Leaves; IS, immature seed nut; SC, seed coat; SN, seed nut; WB, wood (from branches). ^B^Storage of plant materials prior to extraction. Dry, Air dried; FF, fresh-frozen. ^C^Extraction process. D, decoction in water; ME, maceration in 80% ethanol; MO, maceration in rapeseed oil; SW, sonicated in water. ^D^The concentration of ginkgolic acid C15:1 is shown as mean ± SD (of three replicates experiments) in mg compound per gram of dried plant material. NT, Not tested. “-,” No compounds detected. Samples were examined by TLC and HPLC and compared to the following standards: ginkgolide A (1), ginkgolide B (2), bilobalide (3), quercetin (4), kaempferol (5), isorhamnetin (6), rutin (7), ginkgolic acid C15:1 (8).*

**FIGURE 6:**
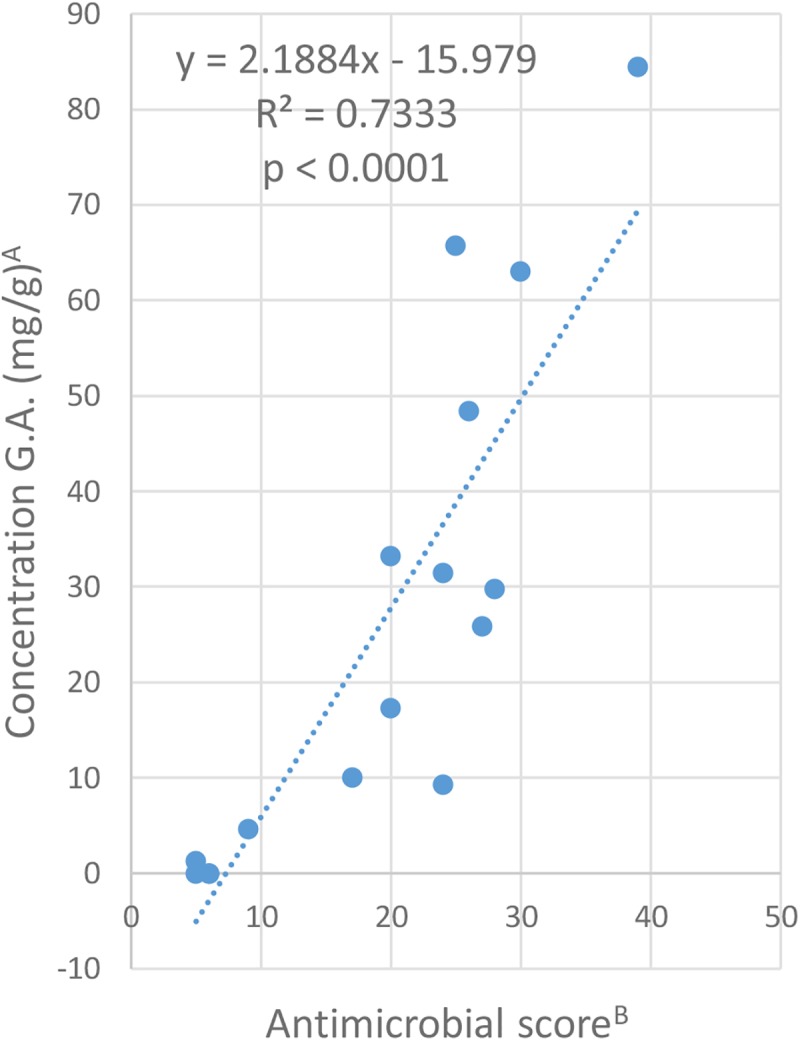
Relationship between the content of ginkgolic acid C15:1 in each extract and their antimicrobial activity. ^A^The concentration of ginkgolic acid C15:1 is shown in mg compound per gram of dried plant material. ^B^Antibacterial score was calculated as follows: Score = [(nb of IC_50_ at 512 μg/mL)^∗^1 + (nb of IC_50_ at 256 μg/mL)^∗^2 + (nb of IC_50_ at 128 μg/mL)^∗^3 + (nb of IC_50_ at 64 μg/mL)^∗^4 + (nb of IC_50_ at 32 μg/mL)^∗^5 + (nb of IC_50_ at 16 μg/mL)^∗^6+ (nb of IC_50_ at 8 μg/mL)^∗^7 + (nb of IC_50_ at 4 μg/mL)^∗^8 + (nb of IC_50_ at 2 μg/mL)^∗^9].

A scoring system adapted from Eloff (1999) was employed to rank Ginkgo extracts according to their antimicrobial activities. Each sample which exhibited an IC_50_ for growth was given one point for an IC_50_ value of 512 μg/mL, two points for an IC_50_ value of 256 μg/mL, three points for an IC_50_ value of 128 μg/mL, and so on. The points were then added together for each Ginkgo extract to yield a score. In order to explore the relationship between the concentration of ginkgolic acid C15:1 in each extract and its antimicrobial score, we plotted the regression line for the data set (Figure 6). Linear regression analysis revealed that the concentration of ginkgolic acid C15:1 contributes about 73.3% to the antimicrobial activity against the bacteria tested (*R*^2^ = 0.7333, *P* < 0.0001).

## Discussion

This study aimed to examine the traditional use of Ginkgo seeds as topical treatment for skin disorders for potential antibacterial efficacy. This is the first study to test and compare the antibacterial activity of various Ginkgo seed extracts on skin pathogens.

In this work, we confirmed the ethnomedicinal importance of seeds in the treatment of skin diseases. As reported in the Compendium of Materia Medica *Ben Cao Gang Mu*, only the seeds were used for medicinal uses, especially as a topical treatment for skin infections. Moreover, our study validates the antimicrobial activity of the seed (i.e., seed coats and immature seeds) on three common skin pathogens: *Cutibacterium acnes*, *Staphylococcus aureus* and *Streptococcus pyogenes* by inhibiting their growth and their biofilm formation (for *C. acnes* and *S. aureus*). While the immature seeds have not been studied before, seed coats from *Ginkgo biloba* have previously demonstrated antibacterial activity against other Gram-positive bacteria, such as vancomycin resistant *Enterococcus* spp., *Listeria monocytogenes*, *Listeria innocua*, *Streptococcus pyogenes*, as well as on Gram-negative bacteria including *Escherichia coli*, *Salmonella enterica* serovar Typhimurium and *Shigella dysenteriae* (Choi et al., 2009; Carraturo et al., 2014). Compared to seed nuts, the seed coats and immature seeds exhibited higher inhibitory activities on *C. acnes*, *S. aureus*, and *S. pyogenes* strains. Although seed coats and immature seeds exhibited the highest antibacterial activity against *S. aureus* LAC, the effect of the leaf extract was similar on *S. aureus* UAMS-1, *S. aureus* AH430, *C. acnes*, and *S. pyogenes*. Indeed, Ginkgo leaves have also demonstrated antibacterial activities on various bacteria including *Bacillus subtilis*, *Enterococcus faecalis*, *Escherichia coli*, *Salmonella enterica*, *Staphylococcus aureus* and plant pathogenic bacterial species (Brantner and Grein, 1994; Mazzanti et al., 2000; Tao et al., 2014), and thus we cannot conclude that Ginkgo seeds have better antimicrobial activity on skin pathogens than Ginkgo leaves.

Interestingly, we found a positive correlation between the antimicrobial activity of Ginkgo samples and the concentration of ginkgolic acid C15:1 in each extract. Moreover, compared to the other Ginkgo derived compounds, ginkgolic acid C15:1 exhibited the highest antimicrobial activity profile in our study. Therefore, this compound appears to be involved in the antimicrobial activity of Ginkgo extracts. Ginkgolic acid C15:1 is the most abundant compound from the 6-alkylsalicylic acids with a long-chain hydrophobic base of 13–17 carbons present in *Ginkgo biloba* (Fuzzati et al., 2003; van Beek and Montoro, 2009). The ginkgolic acids have been described to occur in leaves, buds and nutshells of *Ginkgo biloba* at different concentrations. Higher concentrations of ginkgolic acids are found in sarcotesta/seed coats (5.35–12.8%), compared to seed nuts (0.012–0.028%), which corroborates our results (Chan et al., 2007). Ginkgolic acids were reported to possess antibacterial activities on Gram-positive bacteria including *Bacillus amyloliquefaciens*, *Rhodococcus jostii*, *Staphylococcus aureus*, *Streptococcus thermophilus*, and vancomycin-resistant *Enterococcus* spp. Moreover, ginkgolic acids are known to exhibit better antibacterial activity against Gram-positive bacteria than Gram-negative bacteria (Choi et al., 2009; Hua et al., 2017). This is consistent with our study where the Ginkgo extracts are more active on *C. acnes, S. aureus*, and *S. pyogenes* than on *A. baumannii* and *K. pneumoniae*. Other pharmacological effects of ginkgolic acids include antifungal and acaricidal activity which could explain the use of seeds in traditional medicine, especially on crab lice (van Beek and Montoro, 2009).

However, ginkgolic acids have also been reported to be cytotoxic, carcinogenic and genotoxic (Liu and Zeng, 2009; Berg et al., 2015). Regarding skin toxicity, previous studies have demonstrated its role in allergic reactions such as contact dermatitis (Lepoittevin et al., 1989). Moreover, a cytotoxicity assay of a mix of ginkgolic acids was performed on HaCaT cells and the authors found similar results as us (IC_50_ = 21.8 μg/mL) (Hecker et al., 2002). In our study, the TI of ginkgolic acid C15:1 can be considered as low with a range of 0.5–16. One option to reduce the toxicity of this compound is to modify the chemical structure of the molecule (Guo, 2017). As an example, doxorubicin, an anticancer agent isolated from *Streptomyces* species was structurally modified to afford epirubicin with a higher TI (Paul Launchbury and Habboubi, 1993). Regarding antimicrobial compounds, modification of TAN-1057A/B through *de novo* synthesis led to a significant reduction of toxicity (Nussbaum et al., 2006).

Due to their toxicity, the amount of ginkgolic acids in Ginkgo standardized extract is limited at 5 μg/g in the European and U.S. Pharmacopeia, and 10 μg/g in the Chinese Pharmacopeia (Fuzzati et al., 2003; Huang et al., 2017). In the present study, ginkgolic acid C15:1 was found in each Ginkgo extract at more than 1,000 μg/g, and most of the Ginkgo extracts with high concentration (>15 mg/g) in ginkgolic acid were cytotoxic to HaCaT cells. While it is certain that pure ginkgolic acids can lead to skin toxicity, the toxicological impact of Ginkgo extracts remains controversial (van Beek and Montoro, 2009). It was demonstrated that leaf extracts containing approximately 1,000 ppm of ginkgolic acids did not sensitize guinea pigs skin experimentally (Hausen, 1998). Moreover, crude Ginkgo extracts containing 22,000 ppm of ginkgolic acids did not markedly have *in vitro* cytotoxic effects on skin, renal and intestine cells (Siegers, 1999). Finally, in our study, the therapeutic indices of Ginkgo seed coats and immature seeds against *C. acnes*, *S. aureus*, and *S. pyogenes* as compared to a human keratinocyte cell line (HaCats) were above 10, suggesting that these plant extracts could be safely used in the topical applications described in the historic medical text (More et al., 2008). However, further studies should be performed to ensure the safety of Ginkgo seeds in traditional medicine.

Apart from ginkgolic acids, other Ginkgo compounds have also been reported to possess antibacterial activities, and thus could be potentially involved in the activity of our extracts. Flavones (i.e., quercetin, kaempferol and isorhamnetin) exhibited activity against *S. aureus* in our study and in previous studies (Hirai et al., 2010; Teffo et al., 2010; Jiang et al., 2016). However, these compounds are known to be present in only small amounts in Ginkgo leaves, and they were not found in any active Ginkgo extracts from our study. Synergism of all three flavone aglycones should also be taken into consideration since this action was demonstrated on *Bacillus subtilis*, *Micrococcus roseus*, *Pseudomonas putida*, and *Serratia marcescens* (Sati et al., 2018). Numerous flavonol glycoside derivatives of the aglycones quercetin, kaempferol and isorhamnetin have also been identified in Ginkgo extracts and represent 22–27% of the total content of the standardized extract EGb761 (Chan et al., 2007). Although no studies aimed to evaluate their antibacterial activity, other quercetin glycosides have been shown to possess antimicrobial activities (Cushnie and Lamb, 2005).

*Ginkgo biloba* polyprenols (GBP) have also attracted much attention due to their antimicrobial activity. GBPs were shown to possess antimicrobial activity against *Salmonella enterica*, *Staphylocococus aureus*, *Aspergillus niger*, *Escherichia coli*, and *Bacillus subtilis* (Tao et al., 2013). Moreover, GBPs have demonstrated a capacity to enhance the antibacterial activity of antibiotics (Tao et al., 2016). Due to their highly apolar chemical character, it’s unlikely that these compounds could be responsible for the activity of our extracts.

Regarding Ginkgo compounds with activity on the skin microflora, ginkgolide A and ginkgolide B have already demonstrated antimicrobial activity against *Streptococcus pyogenes*, while bilobalide was shown to inhibit the growth of *S. pyogenes* and *Staphylococcus epidermidis* (Boonkaew and Camper, 2005). However, none of these compounds exhibited growth or biofilm inhibitory activity in the skin pathogens studied in our work.

Ginkbilobin, a protein isolated from Ginkgo leaves, also exhibited a moderate antibacterial action against *S. aureus* and *Pseudomonas aeruginosa* (Wang and Ng, 2000).

It should be noted that we focused on the presence of ginkgolic acid C15:1, but we did not examine all ginkgolic acids (i.e., GA13:0, GA15:1, GA17:2, GA15:0, and GA17:1). Although the former is the most abundant one (about 55% of all ginkgolic acids) and it has demonstrated antibacterial activity on various bacterial strains (Fuzzati et al., 2003; Hua et al., 2017), ginkgolic acid C17:1 has also been shown to possess activity against vancomycin-resistant *Enterococcus* spp. and *Staphylococcus aureus* (Choi et al., 2009; Lee et al., 2014). Thus, all ginkgolic acids could act in synergy.

Lastly, among the three aqueous samples which exhibited antibacterial activity (extract number 668, 687, and 689), none of them contained ginkgolic acid C15:1. Further analysis should be performed to isolate the compound or mixture of compounds responsible for this activity.

In summary, our work has validated the traditional use of Ginkgo seeds for treating skin disorders by demonstrating its antibacterial activity against skin pathogens *C. acnes*, *S. aureus*, and *S. pyogenes*. Ginkgolic acid C15:1 was identified as the main compound responsible for the activity in the ethanolic extracts, but its toxicological impact on the skin suggests that further studies should be undertaken to assess the safety of the Ginkgo seeds. In the search for new antibiotics, the structural modification of the ginkgolic acid core could represent one useful strategy for the generation of analogs with potentially reduced toxicity and enhanced specific antibacterial bioactivity.

## Author Contributions

CQ, FC, and JL conceived and designed the experiments. XH, FC, and JL performed the experiments. XH and FC analyzed the data. FC, XH, CQ, and JL wrote the manuscript. All authors helped to finish the manuscript and approved the final manuscript.

## Conflict of Interest Statement

The authors declare that the research was conducted in the absence of any commercial or financial relationships that could be construed as a potential conflict of interest.
