# Supplementary material for: Validation of a 16th Century Traditional Chinese Medicine Use of Ginkgo biloba as a Topical Antimicrobial
Source: Front Microbiol. 2019 Apr 16;10:775. doi: 10.3389/fmicb.2019.00775 (PMC6478001; doi:10.3389/fmicb.2019.00775)
Supplement: Supplementary file 1 [file Table_1.DOCX]

**Figure S1:** Details of the reference related to skin diseases for *Ginkgo biloba* in the *Ben Cao Gang Mu*


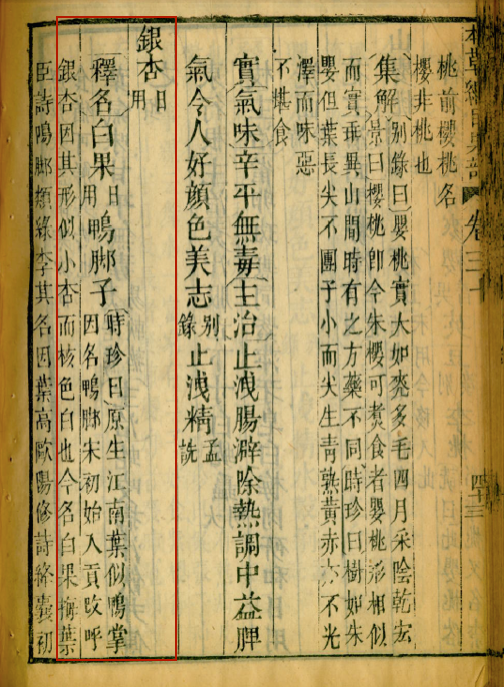


“**Ginkgo (*yin xing*)** (daily use)

**Explanation of its name**[alternate name] **White Fruit (*Bai guo*)** (daily use) [other name] **Duck Foot**

Shizhen commented: [Ginkgo] originated in Jiangnan (region south of Yangtze River). Its leaves look like duck flipper, so it got its nickname: the Duck Foot. Starting from Song Dynasty, Ginkgo was a tribute to royal families and was re-named the silver apricot (*yin xing*), because it looks like small apricot and its inner nutshell is white. Nowadays, it is called the white fruit (*bai guo*).


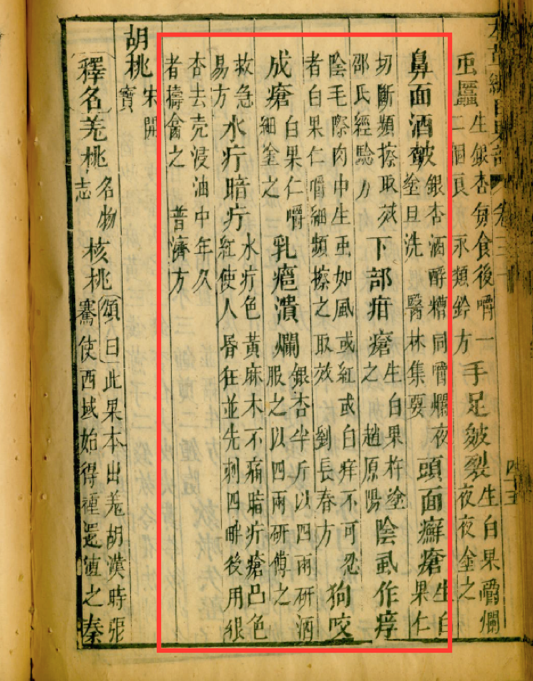


**Chapped hands and feet, rosacea:** Chew up Ginkgo and distillery draff, apply in the evening and wash away in the morning. (*Collection of medicinal reports*)

**Patches and nodules on face and scalp:** cut open Ginkgo kernel, rub on affected parts. (*Remedies out of experience*, by Shao)

**Genital ulcers:** pestle raw Ginkgo and apply on affected parts.(by Zhao Yuanyang)

**Crab louse induced itchiness:** itchiness cause by insects like louse, white or red, grows in skin under pubic hairs. Kernel of Ginkgo, chew up and rub on affected area.

**Dog bite wound abscess:** Kernel of Ginkgo, chew up and rub on affected area.

**Mastitis:** half *jin* (about 250 grams) of Ginkgo, half pestle with wine and drink, the other half pestle [with water]* and apply on affected parts. (*Simple remedies for emergencies, jiu ji yi fang*)

**Bullae or pustules:** furuncles with fluid are yellow, and cause numbness but not pain; dark color furuncles are protruding and red, and cause confusion and madness. Pierce their edge then [apply]6 the paste of Ginkgo that was de-shelled and immersed in oil for a long time. (*General remedies, pu ji fang*)

*The character for water, shui, was missing, but was recorded in the original reference *jiu ji yi fang*.

**Figure S2.** Chemical structures of Ginkgo derived compounds used in this study

**Figure S3.** Represented HPLC chromatograms at 245 nm for all active Ginkgo extracts.

















**Figure S4.** Graphs of data analysis on factors that contribute to effectiveness of Ginkgo extracts.

(A) ANOVA analysis of extraction method (*P* < 0.0001); (B) ANOVA analysis of Ginkgo tree parts (*P* < 0.0001); (C) unpaired t-test of preparation method (*P* = 0.1988); (D) unpaired t-test of gender of the tree collected (*P* = 0.6452).

**Figure S5.** Comparison of extraction methods in MIC assays.

Percent inhibition of extraction methods on growth activity of *A. baumannii* EU27 (A), *K. pneumoniae* EU32 (B), *C. acnes* ATC6919 (C), *S. aureus* UAMS1 (D), *S. aureus* AH1263 (LAC) (E), and *S. aureus* AH430 (F) at 512 µg/mL. The significance levels of each comparison were based on the student’s t-test results on the right of each scatter plots. * p ≤ 0.05, ** p ≤ 0.01, *** p ≤ 0.0001, **** p ≤ 0.00001.

**Figure S6.** Comparison of tree parts in cytotoxicity assays.

Student’s t-test results were also included in the right of the scatter plots of percentage cytotoxicity at highest tested concentration. ** p ≤ 0.01, *** p ≤ 0.0001.

**Figure S7**. Calculation of Limit of Detection (LOD) and Limit of Quantification (LOQ) for the HPLC quantification of ginkgolic acid C15:1 (G.A.)

| **Ginkgolic Acid C15:1 (µg)** | **Peak Area (average)^*^** | **Peak Area (predicted)^**^** | **Residuals (r)^***^** | **r²** |
| --- | --- | --- | --- | --- |
| 0.5 | 531.7666667 | 523.361 | 8.405666667 | 70.6552321 |
| 1 | 1099.833333 | 1079.711 | 20.12233333 | 404.908299 |
| 2 | 2158.233333 | 2192.411 | -34.17766667 | 1168.1129 |
| 4 | 4377.133333 | 4417.811 | -40.67766667 | 1654.67257 |
| 6 | 6700.666667 | 6643.211 | 57.45566667 | 3301.15363 |
| 12 | 13307.2 | 13319.411 | -12.211 | 149.108521 |
|  |  |  | Σr² | 6748.61115 |
|  |  |  | Σr²/(n-1) | 1349.72223 |
|  |  |  | √Σr²/(n-1) **= σ** | 36.738566 |
|  |  |  | **m (slope)** | 1112.7 |
|  |  |  | **LOD** (3.3σ/m) | 0.10895773 |
|  |  |  | **LOQ** (10σ/m) | 0.33017494 |

*Peak area averages were obtained based on the value of the three replicates

**Peak area predicted values were calculated by using the trendline equation from the standard curve

***Residuals were calculated by difference of the predicted value and the observed value

n = number of residuals values

**Table S1.** Description of bacterial strains used in this study

| Species | Strain ID | Characteristics | Resistance profile | Source | Ref. |
| --- | --- | --- | --- | --- | --- |
| *Acinetobacter baumannii* | EU25; OIFC032; NR17778 | Isolate from the wound of a patient in Germany. GenBank: AFCZ00000000 | CFP, CFX, CFZ, PIP | BEI |  |
| *Acinetobacter baumannii* | EU26; OIFC109; NR17780 | Isolate from the residual limb wound of a patient in the US. GenBank: ALAL00000000. | CFD, CFP, CFX, CFZ, GEN, LEV, PIP, SXT, TET, TOB | BEI |  |
| *Acinetobacter baumannii* | EU27; OIFC143; NR17781 | Isolate from the thigh wound of a patient. GenBank sequence: AFDL00000000 | AMP, CFD, CFP, CFX, CFZ, GEN, PIP, SXT | BEI |  |
| *Klebsiella pneumoniae* | EU32; NR15410 | Isolate contains the ß-lactamase *K. pneumoniae* carbapenemase gene (*bla*_KPC_). | AMC, AMP, AZT, CFD, CFN, CFP, CFX, CFZ, ERT, LEV, MER, NIT, PIP, SXT, TZP | BEI |  |
| *Klebsiella pneumoniae* | CDC0016 | Strain with large susceptibility profile. | AMP, FOX, TET | CDC |  |
| *Klebsiella pneumoniae* | CDC0049 | Strain multiresistant, contains carbapenemase. | AMI, AMP, AZT, CFZ, CFP, CFT, FOX, TAZ, TRI, CIP, DOR, ERT, IMI, GEN, LEV, MER, SXT, TET, TIG, TOB, TZP. | CDC |  |
| *Propionibacterium acnes* | ATCC® 6919^TM^, NCTC737 | Type strain. Produces LL-diaminopimelic acid. | MTZ | ATCC |  |
| *Staphylococcus aureus* | UAMS-1, ATCC®49230^TM^ | Osteomyelitis clinical isolate; prototype biofilm isolate. Methicllin sensitive. | BEN | MS | [1] |
|  | UAMS-929 | ΔsarA mutant of UAMS-1 |  | MS | [2] |
|  | AH430 | SA502a + pDB59 cm^R^; agr type II YFP reporter | BEN, CHP | AH | [3] |
|  | LAC, AH1263 | USA300. Methicillin resistant. | BEN, CIP, LEV, MET, MOX, OXA, PIP | AH | [4] |
| *Streptococcus pyogenes* | EU20, MGAS15252, NR33709 | Isolate from the thigh of a patient with an invasive soft tissue infection in Canada. M59 serotype. GenBank: CP003116 |  | BEI |  |
|  | EU21, MGAS9882, NR15272 | Isolate from a patient with a soft tissue infection in Canada. M3 serotype. |  | BEI |  |

**Source:** AH: A. Horswill; ATCC: American Type Culture Collection; BEI: Biodefense and Emerging Infections Research Resources Repository; CDC: Centers for Disease Control and Prevention; MS: M. Smeltzer, UAMS.

**Abbreviations:** AMC, amoxicillin-clavulanic acid; AMI, amikacin; AMP, ampicillin; AZT, aztreonam; BEN, benzylpenicillin; CFD, ceftazidime; CFN, cefoxitin, CFP, cefepime; CFT, cefotaxime; CFX, ceftriaxone; CFZ, cefazolin; CHP, chloramphenicol; CIP, ciprofloxacin; DOR, doripenem; ERT, ertapenem; FOX, cefoxitin; GEN, gentamicin; IMI, imipenem; LEV, levofloxacin; MER, meropenem; MET, methicillin; MOX, moxifloxacin; MTZ, metronidazole; NIT, nitrofurantoin; OXA, oxacillin; PIP, piperacillin; SXT, trimethoprim-sulfamethoxazole; TAZ, ceftazidime; TET, tetracycline; TIG, tigecycline; TOB, tobramycin; TZP, piperacillin-tazobactam.

**References:**

1. Gillaspy AF, Hickmon SG, Skinner RA, Thomas JR, Nelson CL, Smeltzer MS: **Role of the accessory gene regulator (agr) in pathogenesis of staphylococcal osteomyelitis**. *Infect Immun* 1995, **63**(9):3373.

2. Beenken KE, Blevins JS, Smeltzer MS: **Mutation of *sarA* in *Staphylococcus aureus* Limits Biofilm Formation**. *Infect Immun* 2003, **71**(7):4206.

3. Kirchdoerfer RN, Garner, A.L., Flack, C.E., Mee, J.M., Horswill, A.R., Janda, K.D., Kaufmann, G.F., Wilson, I.A.: **Structural basis for ligand recognition and discrimination of a quorum-quenching antibody**. *Journal of Biological Chemistry* 2011, **286**:17351-17358.

4. Boles BR, Thoendel M, Roth AJ, Horswill AR: **Identification of Genes Involved in Polysaccharide-Independent Staphylococcus aureus Biofilm Formation**. *PLOS ONE* 2010, **5**(4):e10146.

**Table S2.** Results of the HPLC quantification of ginkgolic acid C15:1 in each active Ginkgo extract

| **Extracts** | **Mass extract (g)** | **Peak Area 1^*^** | **Conc. G.A. 1 (mg/g)^**^** | **Peak Area 2^*^** | **Conc. G.A. 2 (mg/g)^**^** | **Peak Area 3^*^ (mg/g)** | **Conc. G.A. 3 (mg/g)^**^** | **Average conc. G.A.^***^ (mg/g)** | **Std dev^****^** |
| --- | --- | --- | --- | --- | --- | --- | --- | --- | --- |
| 660 | 0.0002 | 3606.7 | 16.35921288 | 3959.5 | 17.95049567 | 3906.4 | 17.69691102 | 17.33553986 | 0.854977819 |
| 663 | 0.0004 | 506.3 | 1.246690519 | 569 | 1.336233778 | 575.7 | 1.349581232 | 1.310835176 | 0.055950348 |
| 665 | 0.0002 | 6272.2 | 28.28005367 | 6784.5 | 30.68030822 | 6787.9 | 30.67196506 | 29.87744231 | 1.383385438 |
| 666 | 0.0002 | 7182.4 | 27.51725083 | 2094.2 | 27.17978341 | 2009.8 | 27.01801474 | 27.23834966 | 0.254718812 |
| 667 | 0.0002 | 3982.6 | 36.08067979 | 3497.9 | 31.74093367 | 3526.7 | 31.97433357 | 33.36531568 | 2.440968082 |
| 670 | 0.0001 | 7388.6 | 66.54579606 | 7233.4 | 65.40621846 | 7307.8 | 66.02602666 | 65.99268039 | 0.570520167 |
| 671 | 0.0001 | 2241.1 | 10.25187835 | 2235.7 | 10.18283165 | 2162.4 | 9.843885086 | 10.09286503 | 0.218369231 |
| 674 | 0.0002 | 979.1 | 4.607871199 | 1011.4 | 4.665978731 | 1023.8 | 4.716903818 | 4.663584583 | 0.054555724 |
| 677 | 0.0002 | 10829.1 | 48.65974955 | 10784.2 | 48.70346972 | 10727.6 | 48.41196866 | 48.59172931 | 0.157204586 |
| 678 | 0.0002 | 14879 | 66.77200358 | 14031.1 | 63.33441781 | 13279 | 59.9006214 | 63.33568093 | 3.435691264 |
| 679 | 0.0002 | 7210 | 32.47415027 | 6867.8 | 31.05566871 | 6897.4 | 31.16503062 | 31.56494987 | 0.789287043 |
| 680 | 0.0002 | 5952.4 | 26.84982111 | 5659.1 | 25.60911139 | 5599 | 25.31848883 | 25.92580711 | 0.813306074 |
| 683 | 0.0001 | 9312.5 | 83.75420394 | 9410.3 | 85.025 | 9481.2 | 85.59915346 | 84.7927858 | 0.944141018 |

*Peak area values were obtained from chromatograms at 245 nm for each triplicate

**Concentration of ginkgolic acid C15:1 (G.A.) in each triplicate of each Ginkgo extract. This value was calculated by reporting the peak area on the standard curve with ginkgolic acid C15:1 at six different concentrations (see below).

***The average concentration of ginkgolic acid C15:1 (G.A.) was calculated for the three replicates.

****The standard deviation of the concentration of ginkgolic acid C15:1 (G.A.) was calculated for the three replicates.

Formula of the regression line are:

- **replicate 1:** y = 1118x - 51.22 ;

R^2^ = 0.9999

- **replicate 2:** y = 1109.6x - 24.074 ; R^2^ = 1
- **replicate 3:** y = 1110.4x – 23.73 ; R^2^ = 0.9999
